# Gastrointestinal stromal tumor in the fourth portion of the duodenum does not express the CD117: A case report

**DOI:** 10.1016/j.amsu.2022.103560

**Published:** 2022-03-29

**Authors:** Marouane Harhar, Abdelhakim Harouachi, Nada Akouh, Abderrahmane Atmani, Houssam Aabdi, Tariq Bouhout, Amal Bennani, Badr Serji, Tijani EL Harroudi

**Affiliations:** a Surgical Oncology Department, Regional Oncology Center, Mohammed VI University Hospital, Oujda, Morocco; b Mohammed First University Oujda, Faculty of Medicine and Pharmacy Oujda, Oujda, Morocco; c Department of Pathology, Mohammed VI University Hospital, Oujda, Morocco

**Keywords:** Gastrointestinal stromal tumors, The fourth portion of the duodenum, CD117, PDGFRA mutation

## Abstract

**Introduction:**

Gastrointestinal stromal tumors (GISTs) are the most common mesenchymal tumors of the digestive tract.

**Presentation of case:**

54-year-old woman with a history of phyllodes tumor of the left breast he patient was admitted to our hospital for management of retroperitoneal soft tissue tumor, attached to the fourth portion of the duodenum, opposite the head of the pancreas. The patient underwent a large excision of the tumor, the duodenojejunal flexure, and the third and fourth portions of the duodenum along with the head of the pancreas. The histopathological examination confirmed the presence of a spindlecell mesenchymal proliferation. These cells do not express CD117, but they express DOG1. A PDGFRA mutation was identified later. The final diagnosis was duodenal GIST.

**Discussion:**

few cases of GIST in the fourth portion of the duodenum had been reported in the literature. PDGFRA mutation is identified as GISTs tumorigenesis to 15% of cases, and the diagnosis of GISTs is not based solely on the expression of the protein Kit.

**Conclusion:**

the molecular biology examinations are very helpful in the direction of the correct diagnosis in case of negative staining for CD117.

## Highlights

• Few cases of GIST in the fourth portion of the duodenum had been reported in the literature.
• PDGFRA mutation is identified as GISTs tumorigenesis to 15% of cases.
• The molecular biology examinations are very helpful in the direction of the correct diagnosis in case of negative staining for CD117.

## 1 Introduction and importance

Gastrointestinal stromal tumors (GISTs) are the most common mesenchymal tumors of the digestive tract. These entities are arising from the stomach and small intestine in more than 90% of cases. The diagnosis is usually confirmed after the immunohistochemical staining [[1], [2], [3]].

Herein, we report a rare case of GIST arising from the fourth portion of the duodenum, in 54-year-old female patient. We discuss the clinical course and the challenges of diagnosis, and present a brief literature review.

This work has been reported in line with the SCARE 2020 Guideline [4].

## 2 Presentation of case

A 56-years-old female patient presented with three months history of epigastric pain without fever, vomiting, nausea, weight loss or any symptom of gastrointestinal obstruction. The pain was intermittent and relieved by analgesia. She had a history of third grade phyllodes tumor of the left breast treated surgically with adjuvant radiotherapy one year ago. The patient had five vaginal deliveries, and she was a non-smoker with no drugs use. She had no family history of cancer.

Physical examination found epigastric abdominal tenderness in the epigastrium. Her vital signs were otherwise normal. Her laboratory tests, including liver biochemical tests, routine blood examination, hydatid serology, and serum tumor markers, were all within normal limits.

**Fig. 1:**
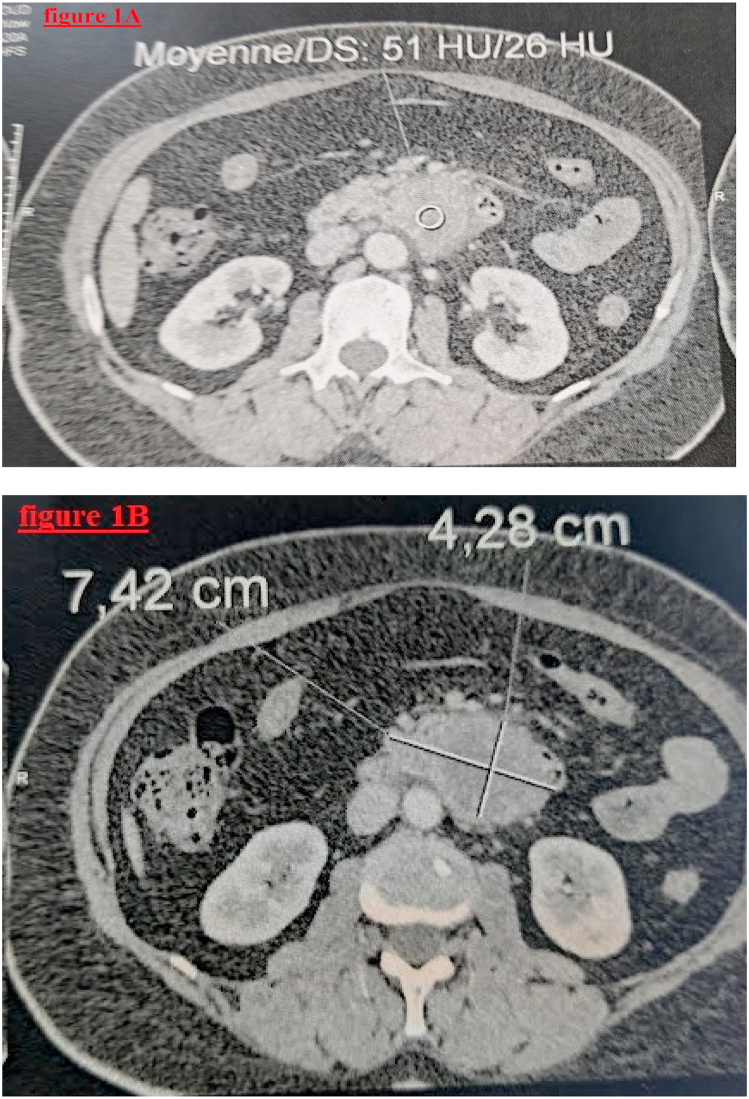
(1A and 1B): MRI shows a retroperitoneal soft tissue tumor, attached to the fourth portion of the duodenum.

Abdominal computed tomography (CT) scan revealed an oval para-jejunal soft tissue tumor, opposite the head of the pancreas, without densification of the adjacent fat nor presence of calcification, measuring 74 x 58 × 43 mm. Magnetic resonance imaging (MRI) confirmed the presence of a retroperitoneal soft tissue tumor, attached to the fourth portion of the duodenum, opposite the head of the pancreas, measuring 63× 55 × 53 mm, suggestive of a stromal tumor (Fig. 1A and B).

These findings were discussed over a multidisciplinary meeting including gastroenterologists, surgeons, radiologists and oncologists. The decision was to proceed with an exploratory laparotomy and excision of the mass.

**Fig. 2:**
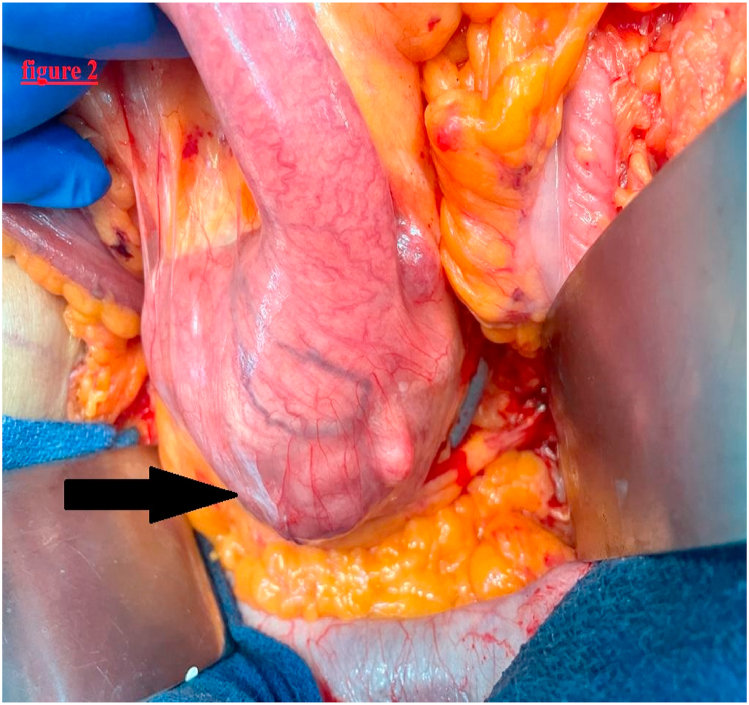
Intra-operative picture of a large mass in the fourth portion of the duodenum (black arrow).

Thus, the patient was operated under general anesthesia, with a midline incision. Intra operatively, A well-circumscribed, soft and cystic mass of the duodenojejunal flexure invading the pancreas was identified. The abdomen and the pelvis were explored without any further abnormal findings. A large excision of the tumor, the duodenojejunal flexure, and the third and fourth portions of the duodenum along with the head of the pancreas was executed without any gross spillage of its containing fluid inside the abdomen, followed by a duodenojejunal anastomosis (Fig. 2). The procedure was achieved by a chief of general surgery, and the operation time was 310 min, and the bleeding amount was 80 ml. a drainage tube was placed next to the anastomosis, and in the Douglas' pouch. The procedure was tolerated well with no early remarkable complication.

**Fig. 3:**
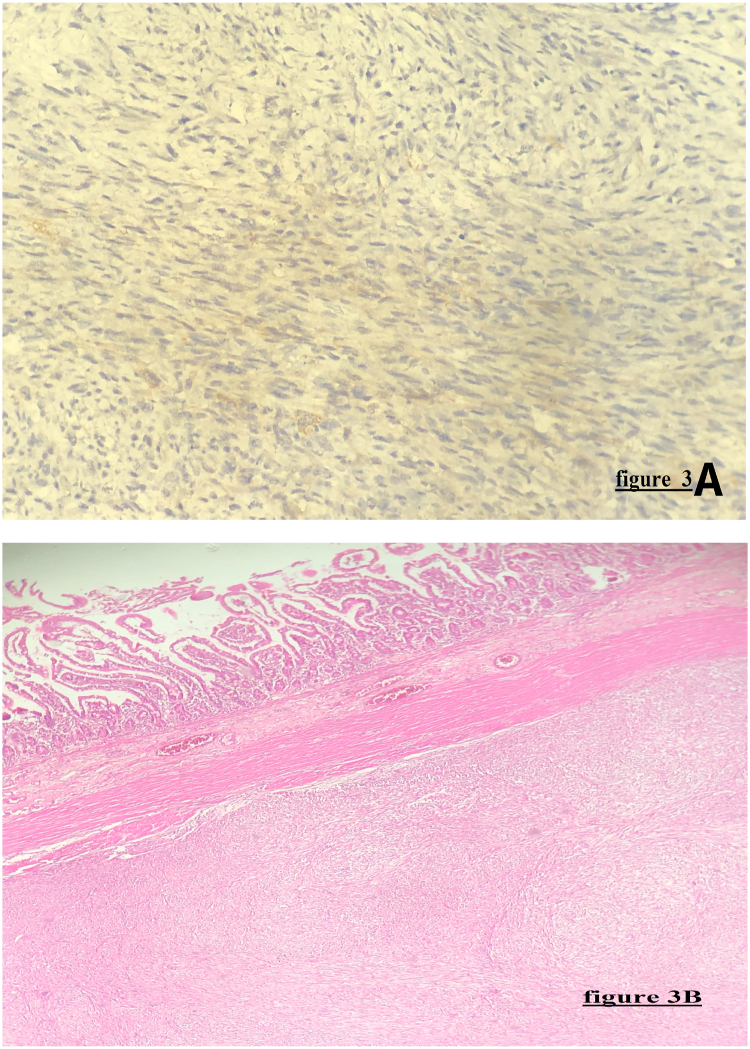
(3A and 3B): Microphotography showing a spindlecell mesenchymal proliferation in the duodenum sub-mucosa (Fig. 3B). the tumor express the DOG1 (Fig. 3A).

Microscopically, the examination of the specimen found a spindlecell mesenchymal proliferation in the duodenum sub-mucosa. Using immunohistochemistery(IHC) staining, the tumor was negative for CD177, CD34, CD44, S100, actin, desmin, myosin, cytokeratin and H-caldesmon. However, a focal and heterogeneous expression of DOG1 and smooth muscle actin was noted (Fig. 3). A PDGFRA mutation was identified later by molecular biology. The final diagnosis was duodenal GIST.

In the postoperative course, the patient suffered from a mild pancreatic fistula (grade A) that was well tolerated. The patient didn't need any intervention for her complication, and was discharged 18 days later. After a follow-up of three months, the patient remains in good health, and there is no recurrence detected.

## 3 Discussion

Gastrointestinal stromal tumors (GISTs) are rare mesenchymal neoplasms located in the digestive tract, recently described [5]. These entities represent the most common soft tissue sarcoma of the gastrointestinal (GI) tract. About 60–70% of GISTs may occur in the stomach. Approximately 30% of GISTs are located in the small intestine, and GISTs of the duodenum originating from the fourth segment are extremely rare. To the best of our knowledge, few cases had been reported until our case study. Zhen Liu et al. reported in their large study that only 22 patients had a primary GIST located in the fourth portion of the duodenum [6]. GISTs are usually diagnosed in the age group of 55–65 years. The estimated incidence of GISTs is 15 cases per million per year, and a total prevalence less than 1% of all gastrointestinal (GI) tumors [7].

Patients with GISTs typically present varying signs and symptoms, varied from non-specific pain to weight loss, GI bleeding (occult or massive), and perforation or a palpable mass. Clinical presentation is widely variable, and depends primarily on the location and size [8]. Duodenal GISTs commonly appear as a regular solid mass. Despite the lack of specific features, radiological exploration (computer tomography scan and MRI) remains essential in the diagnostic approach and post-treatment surveillance [9].

Gastrointestinal stromal tumors (GISTs) result from the anarchic proliferation of the interstitial cell of Cajal (ICC) [10], and they are characterized histopathologically by the almost constant expression of a receptor for a tyrosine kinase called c-kit or CD117 (95% of GIST express C-KIT). The key mechanism involved in GIST pathogenesis is mutation in the proto-oncogene c-Kit [3,8,11]. This genetic modification is present in 86% of cases, and it product the sensitive immunohistochemical marker of the disease: C-Kit (CD117). PDGFRA mutation (the platelet-derived growth factor receptor alpha) is identified as GISTs tumorigenesis to 15% of cases. For that reason the diagnosis of GISTs is not based solely on the expression of the protein Kit. In our case, the patient has been not express CD177, but the PDGFRA mutation was identified [12,13]. Our case exemplifies the challenges of diagnosis of GIST, and clarifies the role of molecular biology in these entities.

The treatment options of GISTs include surgery, and protein-tyrosine kinase inhibitor (Imatinib). Undoubtedly, the ideal treatment for non-metastatic GISTs is complete excision R0 with negative margins, without lymph node excision. The following medical treatment (adjuvant) is imatinib with a median increase in survival approximately 60 months [[13], [14], [15]].

## 4 Conclusion

GISTs of the fourth portion of the duodenum are still extremely rare. Findings from the molecular biology examinations, such as detection of mutations in KIT or PDGFRA are very helpful in the direction of the correct diagnosis in case of negative staining for CD117. The surgical resection is an effective treatment modality. Imatinib therapy 400 mg/day should be indicated for decreasing the risk of recurrence.

## Patient consent

Written informed consent was obtained from the patient for publication of this case report and accompanying images. A copy of the written consent is available for review by the Editor-in-Chief of this journal on request.

## Provenance and peer-review

Not commissioned, externally peer reviewed.

## Ethical approval

No ethical approval necessary.

## Sources of funding

The author(s) received no financial support for the research, authorship and/or publication of this article.

## Author contribution

**Dr Harhar Marouane:** Have written the article, have consulted the patient, prescribed all of the tests and prepared the patient for surgery and participated in the surgery.

**Dr Harouachi Abdelhakim:** have helped writing the article, data collection.

**Dr Akouh Nada:** Interpretation of pathological data.

**Dr Atmani abderrahmane:** have helped writing the article, data collection.

**Dr Aabdi Houssam:** have helped writing the article, data collection.

**Pr Bouhout Tariq** (oncology surgery professor)**:** have supervised the writing of manuscript.

**Pr Bennani Amal** (Pathology professor): confirm the histological diagnosis.

**Pr Serji Badr** (oncology surgery professor): have supervised the writing of the paper.

**Pr El Harroudi Tijani** (oncology surgery professor): Writing, review and editing of the manuscript, and had been the leader surgeon of the case.

## Consent

Written informed consent was obtained from the patient for publication of this case report and accompanying images. A copy of the written consent is available for review by the Editor-in-Chief of this journal on request.

## Trial registry number

Our paper is a case report; no registration was done for it.

## Guarantor

Harhar Marouane.

## Declaration of competing interest

The authors declared no potential conflicts of interests with respect to research, authorship and/or publication of the article.
